# Digital standardization in liver surgery through a surgical workflow management system: A pilot randomized controlled trial

**DOI:** 10.1007/s00423-025-03634-7

**Published:** 2025-03-11

**Authors:** Fabian Haak, Philip C. Müller, Otto Kollmar, Adrian T. Billeter, Joël L. Lavanchy, Andrea Wiencierz, Beat Peter Müller-Stich, Marco von Strauss und Torney

**Affiliations:** 1 Clarunis, Department of Visceral Surgery, University Digestive Health Care Center, St. Clara Hospital and University Hospital Basel, Petersgraben 4, 4031 Basel, Switzerland; 2 Department of Visceral, Transplant, Thoracic and Vascular Surgery, Division of Hepatobiliary Surgery and Visceral Transplant Surgery, University Hospital Leipzig, Leipzig , Germany; 3 Department of General, Visceral, Vascular and Thoracic Surgery, Kantonsspital Baselland, Liestal, Switzerland; 4 Department of Clinical Research, University of Basel, University Hospital, Basel, Switzerland; 5 St. Clara Research Ltd, Basel, Switzerland; 6 Department of Biomedical Engineering, University of Basel, Allschwil, Switzerland

**Keywords:** Surgical procedure manager, Workflow surgery, Liver surgery, Complex surgery

## Abstract

**Introduction:**

Surgical process models (SPM) are simplified representations of operations and their visualization by surgical workflow management systems (SWMS), and offer a solution to enhance communication and workflow.

**Methods:**

A 1:1 randomized controlled trial was conducted. A SPM consisting of six surgical steps was defined to represent the surgical procedure. The primary outcome, termed “deviation” measured the difference between actual and planned surgery duration. Secondary outcomes included stress levels of the operating team and complications. Analyses employed Welch t-tests and linear regression models.

**Results:**

18 procedures were performed with a SWMS and 18 without. The deviation showed no significant difference between the intervention and control group. Stress levels (TLX score) of the team remained largely unaffected. Duration of operation steps defined by SPM allows a classification of all liver procedures into three phases: The Start Phase (low IQR of operation time), the Main Phase (high IQR of operation time) and the End Phase (low IQR of operation time).

**Conclusion:**

This study presents a novel SPM for open liver resections visualized by a SWMS. No significant reduction of deviations from planned operation time was observed with system use. Stress levels of the operation team were not influenced by the SWMS.

**Supplementary Information:**

The online version contains supplementary material available at 10.1007/s00423-025-03634-7.

## Introduction

Surgical quality is determined by attributes belonging to three categories: structure, process and outcome [1, 2]. It is therefore not only dictated by the efforts of the surgeon performing the operation [3, 4]. Communication between team members involved in performing surgery is a key connecting attribute with a large impact on quality [5, 6]. Our previous work showed that surgeons, compared to other high risk professions, do not typically communicate their preoperative plan with other members of the team [7]. Methods like the preoperative team time-out to guarantee communication and thereby improve quality have been developed and are now widely used [8–10]. However, a problem of information overload can exist and therefore an intelligent presentation system is needed to mitigate this. [11–13]

Surgical procedure models (SPM) are simplified representations of a network of surgery-related activities and can be used to overcome infrastructure breaches or communication errors [14]. They can express surgical interventions and thereby provide qualifiable and quantifiable data on the procedural steps making up the operation as a whole. Once defined the model can be fed into a Surgical Workflow Management System (SWMS) that can be used as a visual aid to improve communication and workflow during the operation [15].

The aim of this study was to evaluate if the implementation of a SWMS can improve intraoperative process quality in liver surgery.

## Methods

Ethical approval was sought and a declaration of no objection was provided from the responsible ethics committee of Northwest Switzerland (Project-ID: Req-2020–00249) [16]. The study protocol was registered beforehand with ClinicalTrials.gov under ID NCT04097054.

Reporting of this trial follows the Consort guidelines and the CONSORT 2010 checklist is provided in Supplementary Fig. 1.

### Trial design

A 1:1 randomized controlled study at a single institution was performed to answer the question of whether a SWMS can improve the intraoperative quality of open liver resections. To this end, all patients undergoing an open liver resection during the study period were parallel randomized regarding whether the operating team should use a SWMS (intervention) or not (control) during the operation.

### Participants

All patients receiving an open liver resection at our tertiary center during the trial period were included. Emergency surgery cases and surgery performed on patients younger than 18 years were excluded.

### Randomization

Patients were randomly assigned to intervention group (surgery assisted with SWMS) or to control group (surgery performed without SWMS) based on permuted block (n = 6 per block) randomization using tidyverse sample function in R. Randomization was performed by F.H. and distributed to the supporting team members in the operating theatre tasked with running the SWMS or commencing with control operation. A new randomization block was submitted as soon as the previous one was finished. Patients were unaware as to whether they were treated in the intervention or control arm.

### Interventions

A SPM was defined using a top-down modeling approach. The method is based on the experience of the process modelers and abstracts knowledge to its smaller details [14, 17–19]. Using this technique a preliminary SPM containing 11 categories with a total of 41 sub steps was created to describe the process of open liver surgery. It proved too complicated and was not accepted by the team members performing the operation, so a new generalized model was developed from the initial model [20]. The generalization led to a reduced model containing 6 linear steps (1. Positioning & Installation, 2. Laparotomy, 3. Pre-parenchymal phase, 4. Parenchymal dissection, 5. Post-parenchymal phase, 6. Closure) of the procedure. The initial model and the final model are provided in additional references Figure 2 + 3.

Visualization of the model during the operation was achieved via a SWMS. The SWMS was established based on the SPI® (Surgical Process Institute) platform by Johnson & Johnson Medical GmbH. The SWMS that was used in our case displays a visual and written description of the operation step being performed at that moment. The system additionally shows the next two steps of the operation that follow and visualization is aided by showing a picture of the required instruments for that step to assist instrumentation by the OR nurse. Additionally surgery duration, expected remaining OR time and expected end time were displayed to plan anesthesia care and the following case. The estimation is based on the mean time of the operation steps as calculated from a test cohort. Estimated total duration of the operation is adjusted as soon as deviations from the mean time of the operation steps occur. The SWMS was displayed via a large screen which is centrally placed in the OR to allow clear visibility for all team members. A picture of the SWMS display is provided in additional references Figure 4.

Controls were treated according to the current standard of the institution. Concerning the specific technique of liver resection an extrahepatic glissonian approach was used for inflow control. Major vessels (Left or right portal vein or liver vein) were transected with a vascular stapler. Parenchymal dissection was performed using a CUSA and small ducts were selectively clipped or tied off. In the post-parenchymal phase complete blood hemostasis was achieved with bipolar energy and adjuncts (TachoSil®, Takeda). Additionally drains (Easy-flo®, Dahlhausen) were placed when deemed necessary by the lead surgeon.

### Outcomes

Outcome measures were selected to characterize intraoperative process quality [1, 2].

The primary outcome measure is the absolute difference between actual surgery duration (from skin incision to skin closure) and the planned duration, expressed in minutes per planned hour. The rationale behind this endpoint was that this metric would allow to compare different types of operations in terms of their impact on the entire operating activity in a given unit. It makes longer and shorter operations and different types of procedures comparable, because it offers a relative measure for the precision of preoperative planning on one hand and the performance of different operating team compositions on the other.

The planned duration was determined by the lead surgeon. At the timepoint of determination the lead surgeon was unaware as to which study group would be allocated. Determination was made according to the type of resection that was planned.

$$\frac{\vert actual\;surgery\;duration-planned\;surgery\;duration\vert}{planned\;surgery\;duration}\times60\;minutes\;per\;planned\;hour$$

In the following, the primary outcome will be referred to as “deviation”.

Secondary outcomes include self-reported stress levels of operating team members as measured using the raw NASA Task Load Index (TLX) [21, 22] and frequency plus severity of postoperative complications according to the Clavien-Dindo classification [23].

Additionally, duration of operation sub steps defined by SPM was measured by the SWMS.

### Statistical Analysis

A randomized controlled set up us was chosen to minimize bias. The study was not blind as the cognizant interaction between the management system and the test subjects is needed to achieve an outcome. The sample size was calculated assuming a decrease in deviation by 33%, from 20 to 13.3 min per planned hour. Further assuming normality of the deviation, a total of 36 surgeries (18 in each study arm) are needed to show that the deviation is smaller with the SWMS than without at a 1-sided significance level of alpha = 0.025 [24].

Baseline characteristics were compared between the study arms by means of Standardised Mean Differences (SMDs) [25, 26]. Complexity of open liver surgeries was rated as recommended by Kawaguchi [27].

The comparison of the primary outcome was performed using the Welch t-test at a 1-sided significance level of 0.025. Concerning the secondary outcomes, the self-reported stress levels of operating team members were summarized separately for different types of team members (lead surgeon, first assistant surgeon, OR nurse, anesthetist), for all surgeries and stratified by utilization of the SWMS. Mean differences of the NASA TLX between surgeries performed with and without SWMS, together with 95% Welch confidence intervals for the differences were calculated.

Occurrence of intra- and postoperative complications and their severity according to the Clavien-Dindo classification are reported for all surgeries stratified by utilization of SWMS.

Finally, an additional secondary analysis explores to what extent characteristics of patients (patients with previous abdominal surgery), features of the planned surgery (complexity of surgery) or experience of the operating team (joint experience of lead surgeon and theatre nurse > 10 surgeries) explain differences in the primary outcome, using linear regression models. Models were compared based on their adjusted R^2^ and Akaike Information Criterion (AIC) [28]. As about half of the surgeries were performed by surgeon 1, we performed a post-hoc subgroup analysis to see whether the estimated associations are different for this particular surgeon compared to the other surgeons. We estimated the best models according to AIC and adjusted R2 again including additionally interaction terms between the predictors and a binary variable indicating whether surgeon 1 performed the surgery.

All analyses were performed using R Statistical Software (v4.3.; R Core Team 2023).

## Results

### Participant flow

37 open liver resections were included in the study. 19 patients were operated using a SWMS and 18 patients were operated without using a SWMS. One patient’s operation which was followed with the SWMS was terminated due to unexpected infiltration of central vasculature. This patient was excluded from the analysis. Patient flow chart is provided in additional references Figure 5.

### Recruitment

Patients were recruited between December 7th 2022 and September 7th 2023. Trial was ended as soon as the planned size was reached.

### Baseline data

The study cohort comprised patients scheduled for open liver resections at a tertiary care center, all aged 18 years or older. The surgeries performed during the study period represented a range of complexity levels as classified by the Kawaguchi score, encompassing low, medium, and high-complexity procedures. The majority of patients presented with ASA classifications of 2 or 3, reflecting a typical surgical population with moderate-to-severe underlying health conditions. Both groups underwent planned operations, and their clinical and demographic characteristics were representative of patients undergoing major hepatic resections.

The groups differed in three categories: The intervention group had received previous abdominal operations more often (intervention group 55.6% vs control group 38.9%). Also more patients with an ASA class of 4 were randomized into the intervention group (intervention group 11.1% vs control group 0%). Finally, more patients received a non-anatomic confined resection in the intervention group (intervention group 27.8% vs control group 5.6%). Table 1 summarizes baseline characteristics of the operated patients, the surgical procedures, and the operating team members.

**Table 1:** Baseline characteristics of patients, surgical procedures and operating team members

	Level	Control	Planning tool	SMD
n		18	18	
Gender (%)	male	10 (55.6)	13 (72.2)	0.352
	female	8 (44.4)	5 (27.8)	
Diabetes (%)	no diabetes	16 (88.9)	14 (77.8)	0.302
	insulin	1 (5.6)	2 (11.1)	
	oral medication	1 (5.6)	2 (11.1)	
Previous abdominal surgery (%)	no	11 (61.1)	8 (44.4)	0.339
	yes	7 (38.9)	10 (55.6)	
ASA class (%)	2	0 (0.0)	1 (5.6)	0.632
	3	18 (100.0)	15 (83.3)	
	4	0 (0.0)	2 (11.1)	
ICU stay (%)	no	0 (0.0)	6 (33.3)	1
	yes	18 (100.0)	12 (66.7)	
Resection type (%)	non-anatomic/ metastasectomy	1 (5.6)	5 (27.8)	0.914
	left lateral sectionectomy	3 (16.7)	1 (5.6)	
	segmentectomy	1 (5.6)	2 (11.1)	
	bisegmentectomy (other than seg. 2 + 3)	3 (16.7)	1 (5.6)	
	right hepatectomy	5 (27.8)	4 (22.2)	
	left hepatectomy	4 (22.2)	5 (27.8)	
	extended left hepatectomy	1 (5.6)	0 (0.0)	
Complexity Score (Kawaguchi) (%)	low complexity	3 (16.7)	8 (44.4)	0.659
	medium complexity	9 (50.0)	7 (38.9)	
	high complexity	6 (33.3)	3 (16.7)	
Lead surgeon ID (%)	surgeon 1	11 (61.1)	8 (44.4)	0.873
	surgeon 2	0 (0.0)	1 (5.6)	
	surgeon 3	0 (0.0)	1 (5.6)	
	surgeon 4	1 (5.6)	0 (0.0)	
	surgeon 5	0 (0.0)	0 (0.0)	
	surgeon 6	3 (16.7)	3 (16.7)	
	surgeon 7	1 (5.6)	0 (0.0)	
	surgeon 8	2 (11.1)	5 (27.8)	
Lead surgeon 1 (%)	surgeon 1	11 (61.1)	8 (44.4)	0.339
	other surgeon	7 (38.9)	10 (55.6)	
Joint operations surgeon/nurse (%)	0	0 (0.0)	0 (0.0)	0.781
	1–5	3 (17.6)	3 (16.7)	
	6–10	6 (35.3)	3 (16.7)	
	11–20	3 (17.6)	6 (35.3)	
	21–50	5 (29.4)	3 (16.7)	
	> 50	0 (0.0)	2 (11.8)	
Joint operations surgeon/1st assistant (%)	< 21	10 (55.6)	8 (44.4)	0.452
	21–50	1 (5.6)	0 (0.0)	
	> 50	7 (38.9)	10 (55.6)	
Years experience 1st assistant (median [IQR])		18.00 [16.50, 24.00]	24.00 [18.00, 24.00]	0.29
Years experience nurse (median [IQR])		3.00 [2.00, 8.00]	3.00 [1.00, 10.00]	< 0.001

### Primary Outcome

The mean absolute deviation in the control setting was 19.86 min per planned hour (SD 13.83), while the mean absolute deviation when using SWMS was 19.56 min per planned hour (SD 12.67). Table 2 summarizes the observed data of the absolute deviation and of other outcomes. The null hypothesis of the 1-sided Welch t-test was not rejected at the 2.5% level, as the corresponding p-value was 0.473. The average absolute deviation was 0.3 min per planned hour less when the SWMS was used compared to when it was not used (95% Welch CI [−8.7, 9.3].

**Table 2:** Primary and secondary endpoints

	Level	Control	Planning tool	SMD
n		18	18	
Planned duration (minutes) (mean (SD))		244.94 (47.07)	243.11 (44.48)	0.04
Actual duration (minutes) (mean (SD))		211.61 (82.58)	197.28 (82.56)	0.174
Absolute deviation (mean (SD))		19.86 (13.83)	19.56 (12.67)	0.023
Post-operative complication (%)	no	6 (33.3)	8 (44.4)	0.229
	yes	12 (66.7)	10 (55.6)	
Post-operative complication category (%)	none	6 (33.3)	8 (44.4)	0.678
	1	1 (5.6)	0 (0.0)	
	2	6 (33.3)	5 (27.8)	
	3a	2 (11.1)	1 (5.6)	
	3b	0 (0.0)	0 (0.0)	
	4a	1 (5.6)	1 (5.6)	
	4b	1 (5.6)	3 (16.7)	
	5	1 (5.6)	0 (0.0)	
Blood loss (mean (SD))		647.22 (540.83)	609.44 (645.68)	0.063
Stress level surgeon (mean (SD))		37.22 (21.66)	42.82 (17.32)	0.286
Stress level 1st assistant (mean (SD))		38.33 (17.21)	37.54 (20.20)	0.042
Stress level 2nd assistant (mean (SD))		36.99 (17.05)	34.63 (17.67)	0.136
Stress level nurse (mean (SD))		27.82 (17.41)	30.81 (17.36)	0.172
Stress level anaesthesist (mean (SD))		37.28 (14.33)	36.86 (8.16)	0.036

**Fig. 1:**
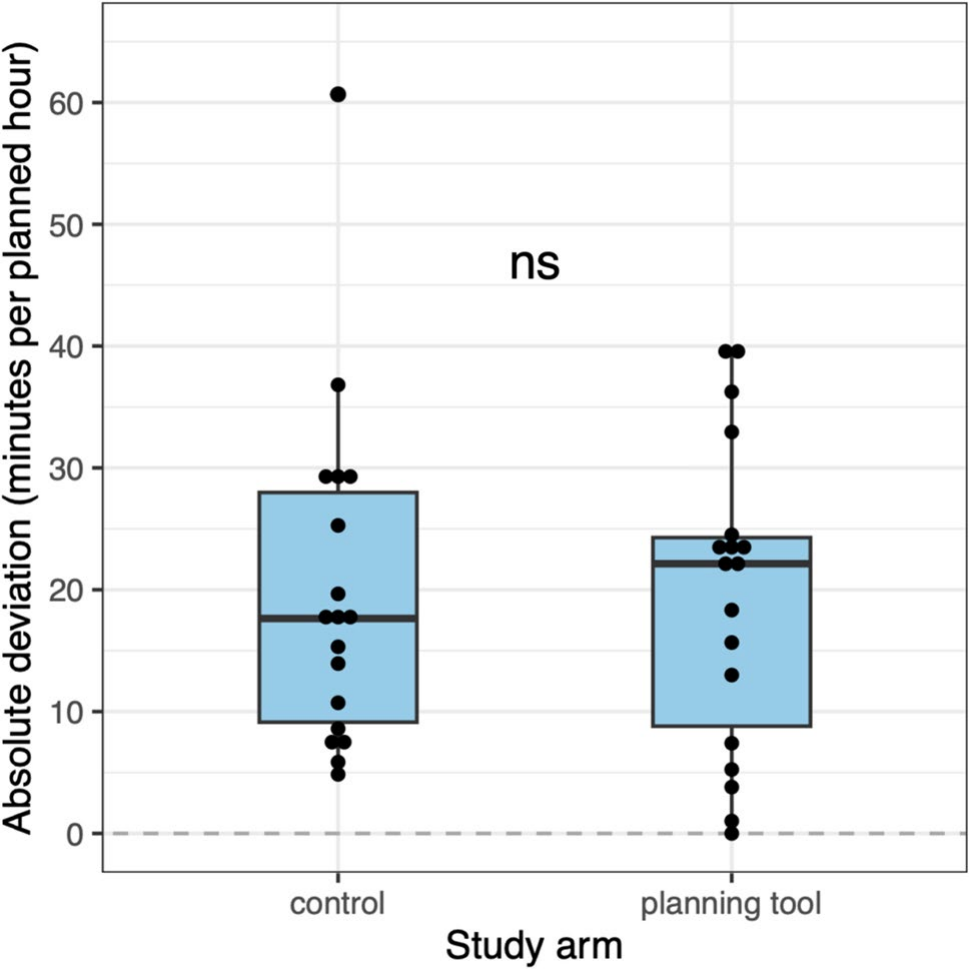
Visualization of primary endpoint, no significant difference between intervention and control group concerning absolute deviation in operation time

Figure 1 illustrates the distribution of absolute deviation by study arm. The observed distributions are slightly skewed but appear to be unimodal. We considered the deviation data as realizations of approximately normally distributed random variables for primary analysis. (Additional references Figure 6).

### Secondary Outcome

**Fig. 2:**
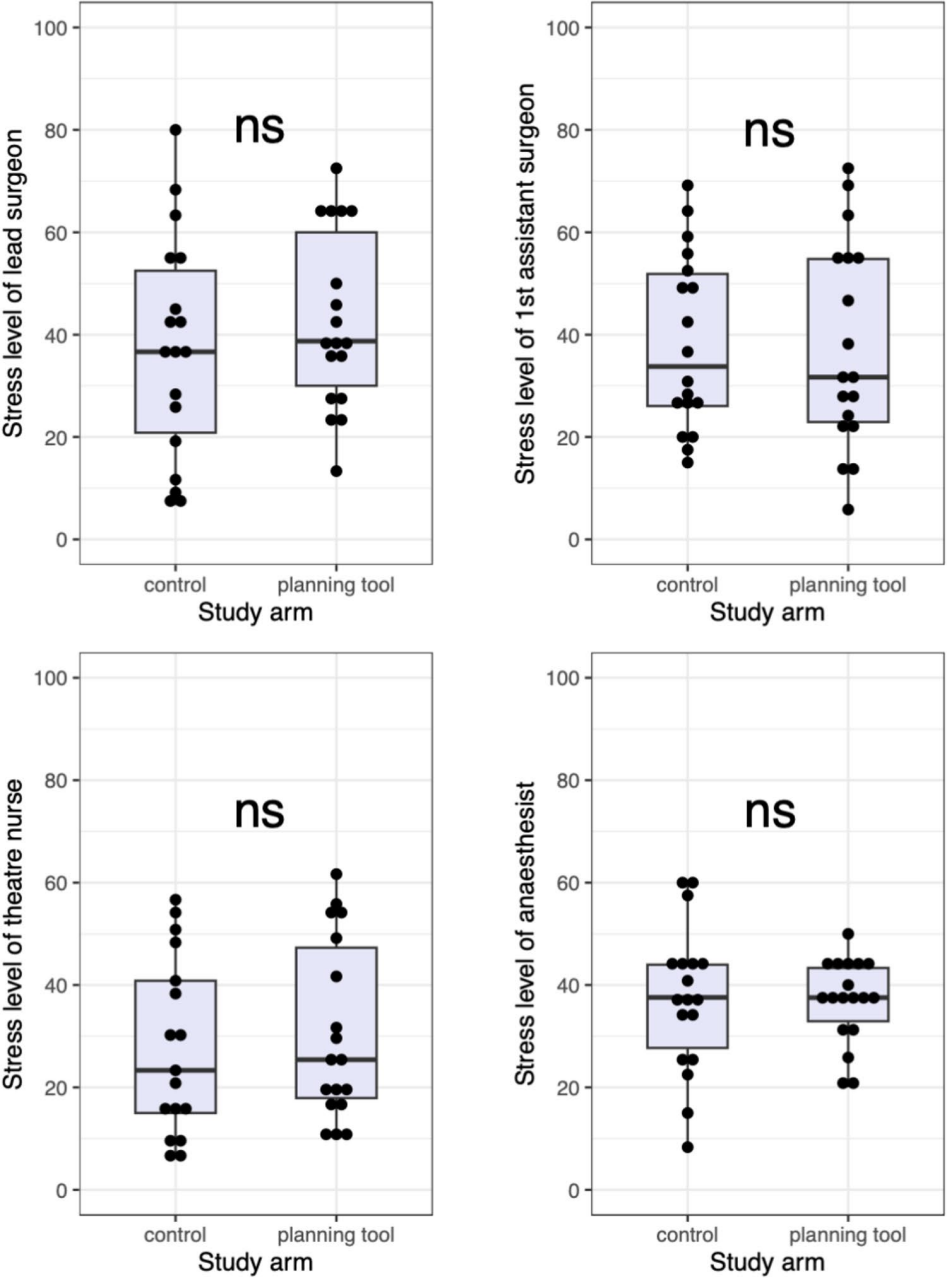
Visualization of TLX stress levels of team members, no significant differences in stress level of team members between intervention and control group

None of the measured differences in secondary outcomes were statistically significant. With regard to the stress levels, the lead surgeon and nurses experienced slightly higher mean stress levels in the intervention group (Lead surgeon: 42.82 vs 37.22, 95% CI for the difference [−18.9, 7.7], Nurses 30.81 vs 27.82, 95% CI [−15.0, 9.0]). Mean stress level for the first assistant and anesthetists were higher in the control group (first assistant: 38.33 vs 37.54, 95% CI [−11.9, 13.5], anesthetist: 37.28 vs 36.86, 95% CI [−7.6, 8.4]). (Fig. 2).

The estimated risk difference of experiencing any post-operative complication is 11.1 percentage points (i.e. proportion of complications “control” group – proportion of complications “SWMS”, 95% Newcombes Hybrid Score CI [−19.2, 38.8]). That is, patients who were operated with the SWMS, had a lower risk (55.6%) of experiencing complications after the surgery, compared to patients operated without the SWMS (66.7%). As an indicator of intra-operative complications, we compared the average blood loss. We found that in surgeries performed with the SWMS, the average blood loss was 37.8 ml lower than when it was not used (95% Welch CI [−366.1 ml, 441.7 ml]).

**Fig. 3:**
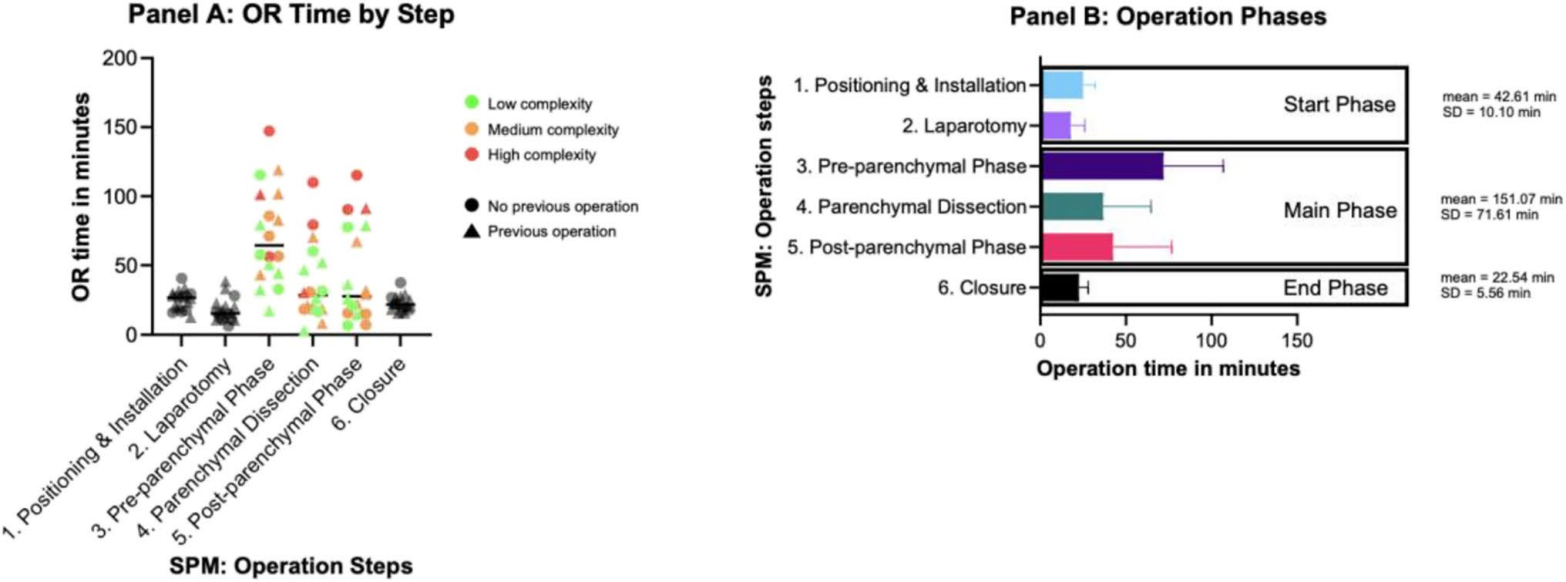
Panel A shows duration of operation steps stratified by operation complexity and if patient received previous operations, Panel B emphasizes standard deviation of operation steps and resulting categorization into three phases

Figure 3 Panel A shows the absolute OR time per OR step stratified by complexity of operation and patients having received previous abdominal operations. Step 1 (mean time = 20.8 min, SD = 5.86 min), 2 (Mean time = 17.6 min, SD = 8.5 min) and 6 (mean time = 22.5 min, SD = 5.6 min) show a high degree of consistency compared to step 3 (mean time = 71.9 min, SD = 34.9 min), 4 (mean time = 35.4 min, SD = 28.2 min) and 5 (mean time = 43.6 min, SD = 34.9 min)

### Secondary analyses

A secondary analysis explored to what extent additional characteristics might explain variability in the primary outcome. We estimated linear regression models for the primary outcome, considering all possible combinations of the 3 variables (previous abdominal surgery, joint experience of lead surgeon and theatre nurse > 10 surgeries and complexity of the planned surgery), in addition to the utilization of the SWMS. Table 3 shows all models together with their adjusted R^2^ and AIC values. The best model in terms of the AIC, was the one considering only the study arm as predictor. The best model in terms of adjusted R^2^ criterion, was the model including previous abdominal surgery as additional predictor.

**Table 3:** Results of linear regression models exploring the impact of certain variables on primary outcome

Model name	AIC	Adjusted R^2
Intervention arm	177.02	−0.0283
Intervention arm + Previous abdominal surgery	177.23	−0.0071
Intervention arm + Joint operation > 10	179	−0.061
Intervention arm + Previous abdominal surgery + Joint operation > 10	179.04	−0.035
Intervention arm + Complexity score	180.96	−0.0952
Intervention arm + Previous abdominal surgery + Complexity score	181.19	−0.0755
Intervention arm + Joint operation > 10 + Complexity score	182.93	−0.132
Intervention arm + Previous abdominal surgery + Joint operation > 10 + Complexity score	182.96	−0.1062

## Discussion

This study introduces a novel SPM for open liver resections. The model was integrated into a SWMS that tracks the surgery being performed.

In our study no significant difference in deviation from planned operation time could be detected when the SWMS was used. The primary endpoint used in this pilot study was not ideally suited to comprehensively analyze the effectiveness of the SWMS in improving surgical quality. As a pilot study, its main purpose was to explore feasibility and inform future research. Moving forward, we plan to adopt a more refined primary endpoint, such as the measurement of workflow disruptions, which is anticipated to provide a more accurate and direct assessment of the system’s impact on surgical quality and efficiency. We plan to focus this analysis especially on the interactions between anesthesia and the surgical team on the one side and the scrub nurses and the surgical team on the other side.

While unaffected by the SWMS, our data highlights a significant disparity between scheduled and actual operation times, averaging 20 min per planned hour of operation. With operations typically lasting an average of 3.5 h, this equates to over an hour of variation per procedure. Considering the imperative of resource optimization, these findings underscore a compelling need to refine planning processes to mitigate the implications of such discrepancies. In future a more intuitive measure of deviation from the planned operation time could simplify the analysis of surgical workflow efficiency. The percentual deviation from planned OR time would, for example, be an option for this measure.

Based on the observation of the standard deviations and interquartile ranges of the duration of the operation steps defined by the SPM we propose three operation phases that define every liver procedure irrespective of procedure complexity and patient characteristics (Fig. 3 Panel B): Phase 1 or Start Phase defined by low standard deviation in duration of steps consisting of SPM Step 1 + 2, Phase 2 or Main Phase defined by high standard deviation in duration of steps consisting of SPM Step 3–5 and Phase 3 or End Phase defined by low standard deviation in duration of step consisting of SPM Step 6.

Future analysis can provide further insight if stratification of the Main Phase steps can lead to an improvement of predictability.

The predictability of Phase 1 and 3 can be invaluable for planning purposes e.g. informing the intensive care unit about arrival and needs of the patient or planning the start of anesthesia for the next patient in the operation room. Possible active interventions by anesthesia team e.g. lowering of central venous pressure or possible blood loss most certainly occur in phase 2 of the operation. A clear predictability of phase 1 can aid in focusing the attention of anesthesia team to critical steps performed in phase 2.

Additionally, our findings do not suggest that the SWMS increases stress levels of the team members. We recorded a slightly higher mean stress level of the lead surgeon and nurse when the SWMS was being used. Confidence interval for the difference in means was rather wide, meaning that differences in both directions are plausible in the light of the data. Interestingly this was the case although the complexity of the operations was lower when the SWMS was being used. Although an a priori power calculation was performed it is possible that a type 2 error exists and that the sample size is too small to reach significant differences in the outcome measures. The impact of the low sample size is highlighted by the wide confidence intervals displayed in our results. Nevertheless operative team members and especially scrub nurses in training did welcome the SWMS as a simple aid to navigate through complex procedures, facilitating staff rotations without extensive prior briefing and training.

The capability of our outcome parameters to measure perioperative quality is a matter of debate. In general measuring surgical quality is not straightforward and conflicting concepts are at play influencing the outcome [29]. As mentioned by Donabedian the “Structure, Process and Outcome” model is a possible approach to quantifying quality in health care [1, 2]. For low-volume, high-risk procedures structural measures are recommended as surrogate parameters for quality assessment [29]. Both our primary and secondary outcomes fall into this category. We acknowledge that our approach is not an exhaustive analysis of the perioperative surgical quality, but we believe that it is a valid one when taking the pertinent literature into consideration. In light of our previous research showing a potential deficit in communication of surgeons with other team members we believe that this method is a valuable approach to addressing this problem [7]. Further studies are needed to address an integration of this surgical procedure model into a perioperative tool kit to achieve quality improvement.

SPMs visualized with SWMS have been successfully introduced into other surgical fields [30, 31]. The fields of surgery in which these SPMs were introduced are more standardized and show less complexity compared to liver surgery. The results of these studies showed that the introduction of SWMS assistance can reduce postoperative complications [31] and the required time slots for operations [30]. This study shows that introduction of SPMs and visualization by SWMS is also possible for more variable, highly complex operations.

Our current study only included open liver resections. This is the case because during the study period this was the predominant technique to perform complex liver resection at our institution. The stable set-up of the SWMS was optimized as surgery was performed in the same location and infrastructure was stable. The model is generalized and can therefore represent laparoscopic or robotic procedures effortlessly and should be expanded to these techniques in future.

This study has limitations. Using a top-down modeling approach the generation of the model was subjective in nature. As such the model cannot claim to be an objectively complete representation of the process. It can have insufficient resolution and exclude aspects that are of importance for the overall process [14]. We believe that we have minimized this problem by including several experts in the generation process.

## Conclusion

This study introduces a novel SPM for open liver resections, visualized through a SWMS. While no significant reduction in deviations from the planned operation time was observed with system use, no safety concerns arose, and it proved valuable for predicting operation end times.

## Supplementary Information

Below is the link to the electronic supplementary material.

Supplementary file1 (DOCX 3734 KB)

## Data Availability

No datasets were generated or analysed during the current study.
