# Supplementary material for: Digital standardization in liver surgery through a surgical workflow management system: A pilot randomized controlled trial
Source: Langenbecks Arch Surg. 2025 Mar 11;410(1):96. doi: 10.1007/s00423-025-03634-7 (PMC11897067; doi:10.1007/s00423-025-03634-7)
Supplement: Supplementary file 1 — Supplementary file1 (DOCX 3734 KB) [file 423_2025_3634_MOESM1_ESM.docx]

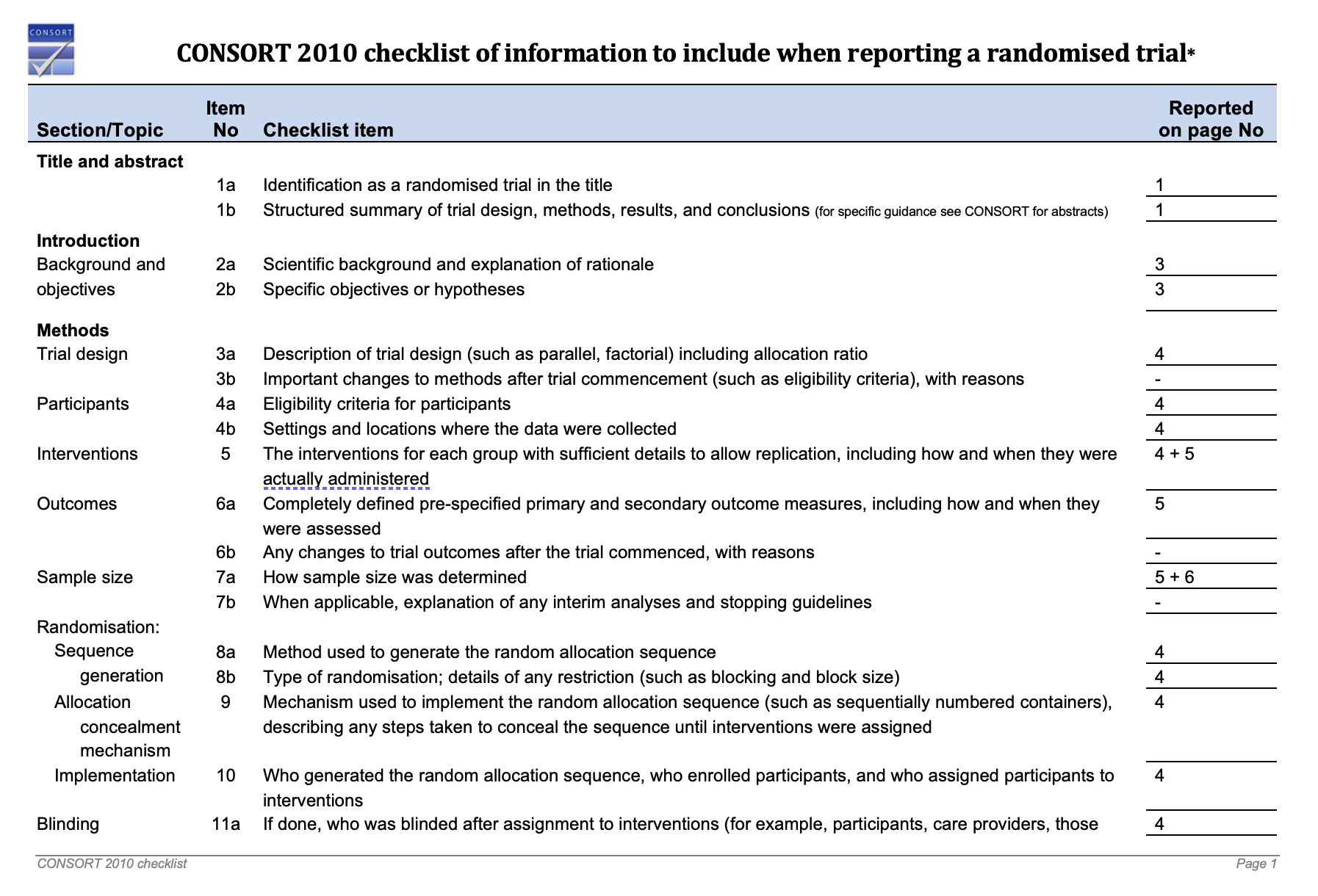


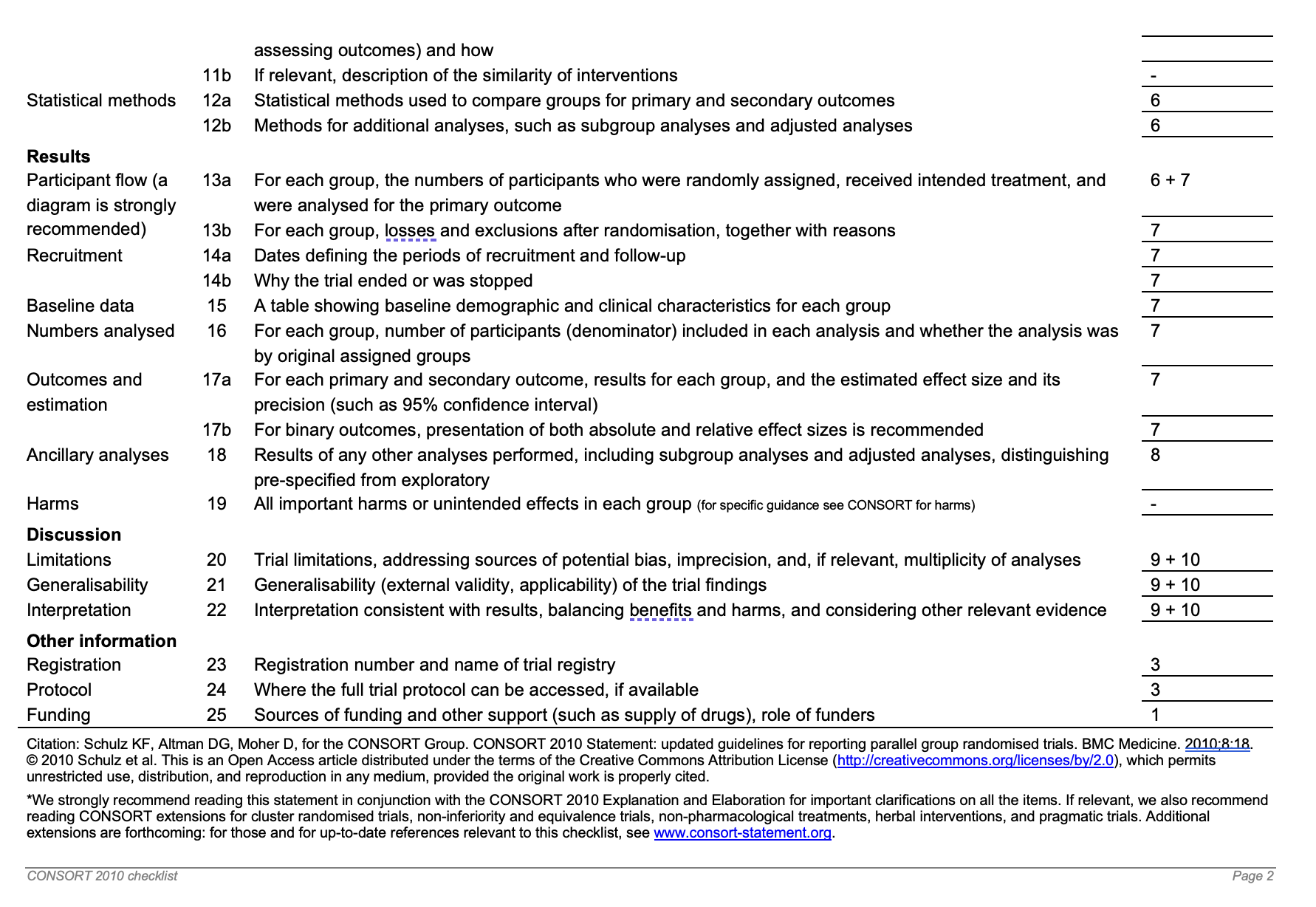


Additional references Figure 1 – Consort 2010 checklist when reporting randomized controlled trial


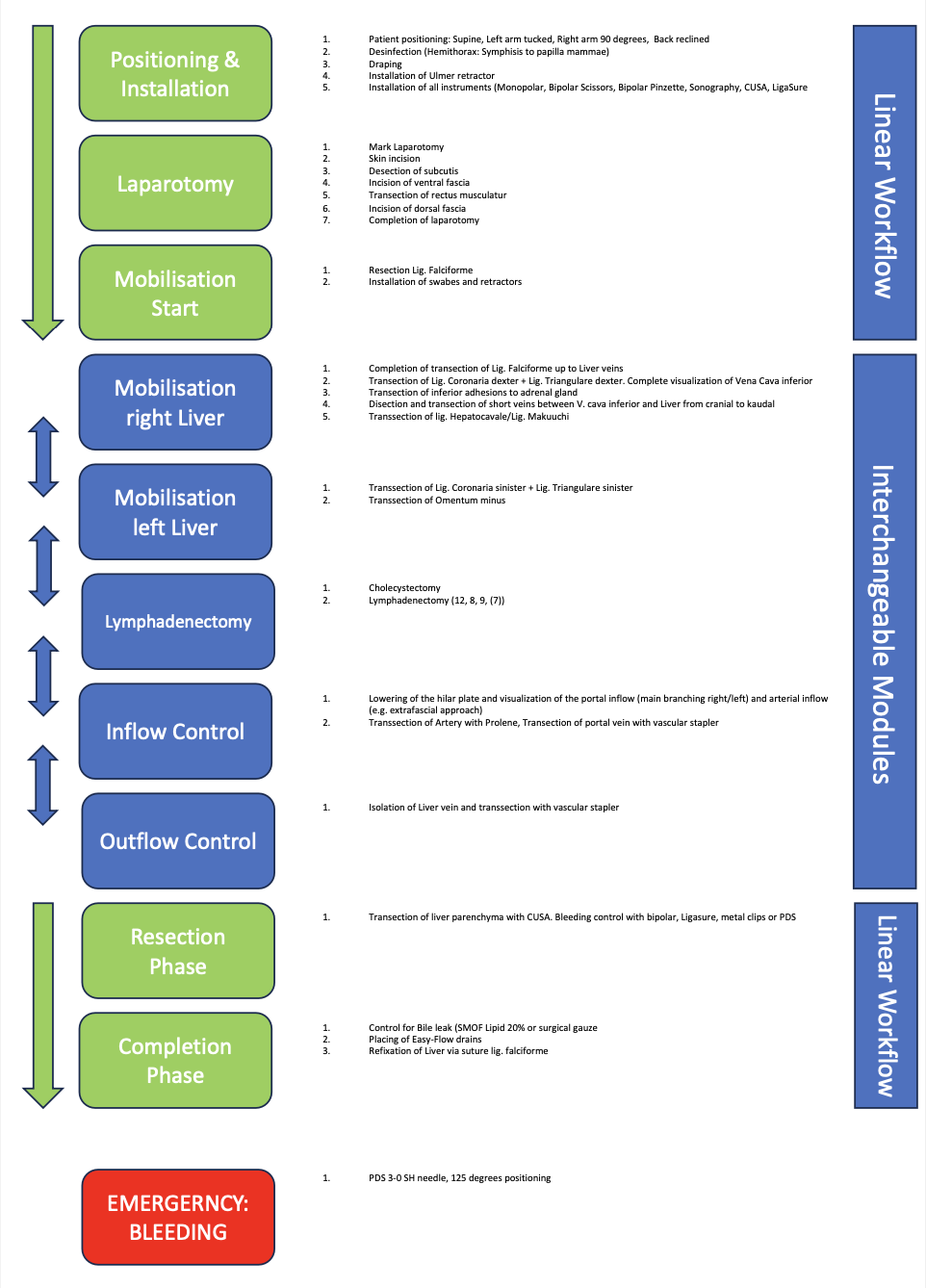


Additional references figure 2 – Initial Surgical Procedure Model


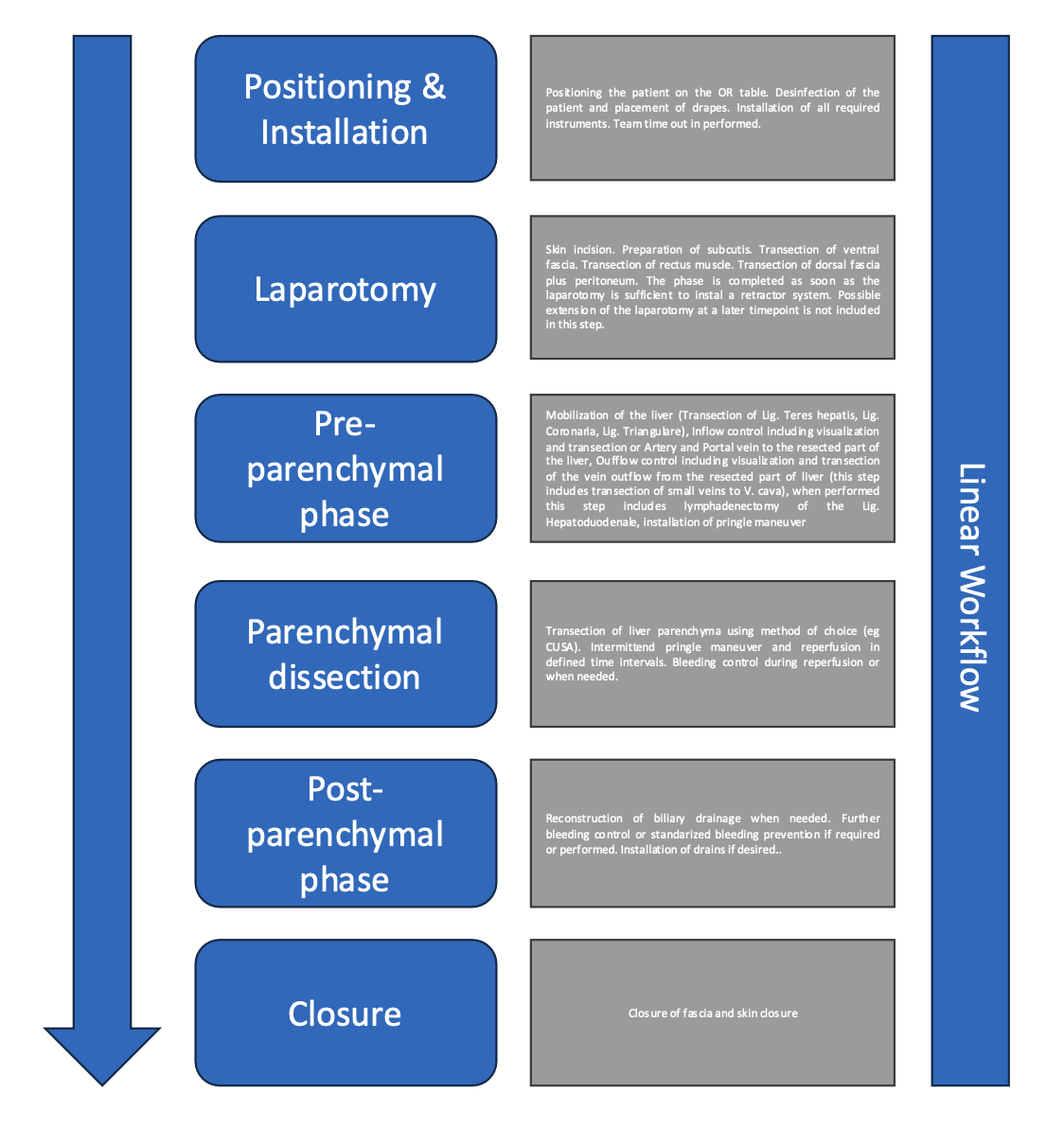


Additional references figure 3 – Final Surgical Procedure Model used after generalization of initial model


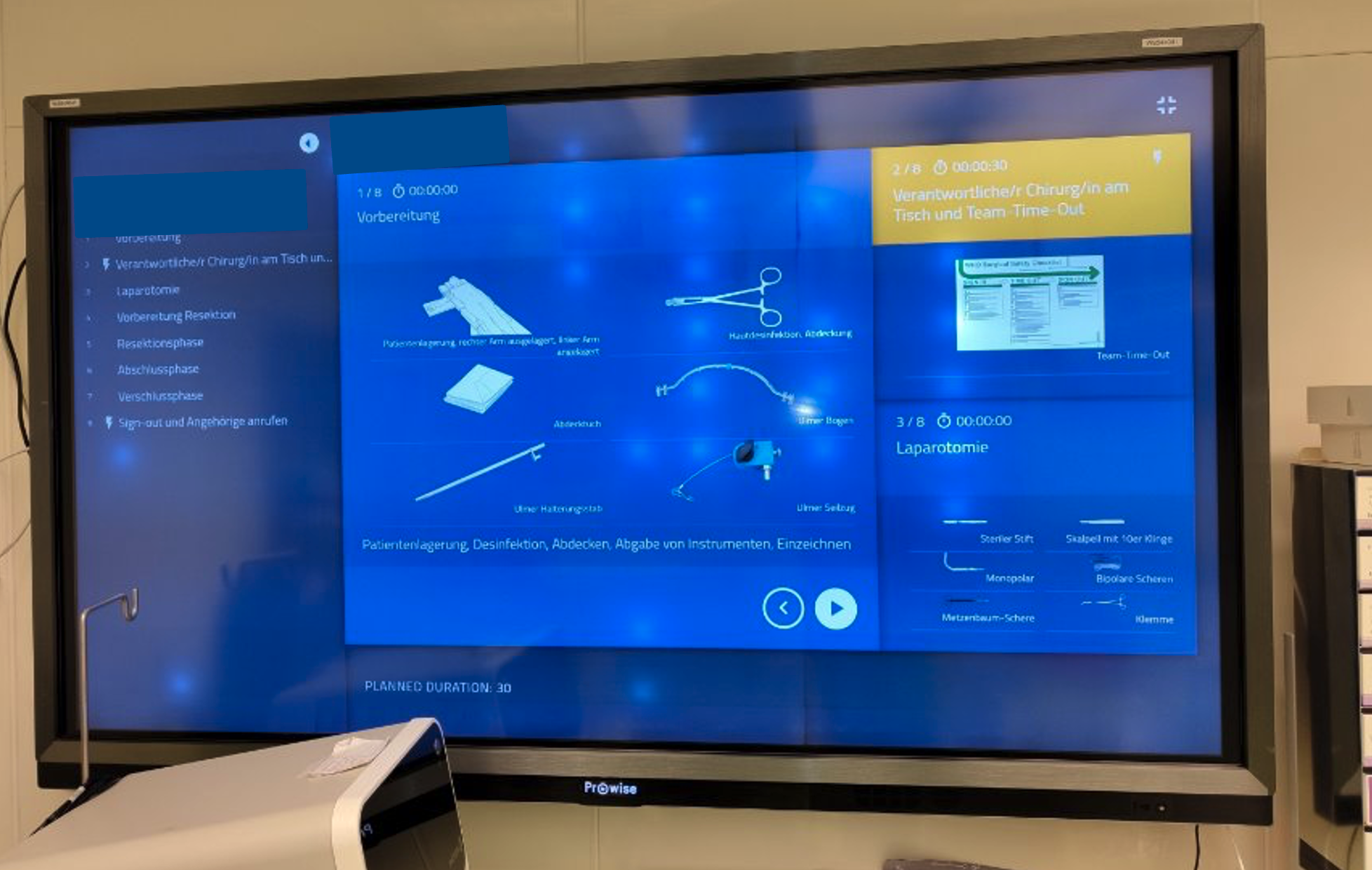


Additional references figure 4 – Display of SWMS in the OR


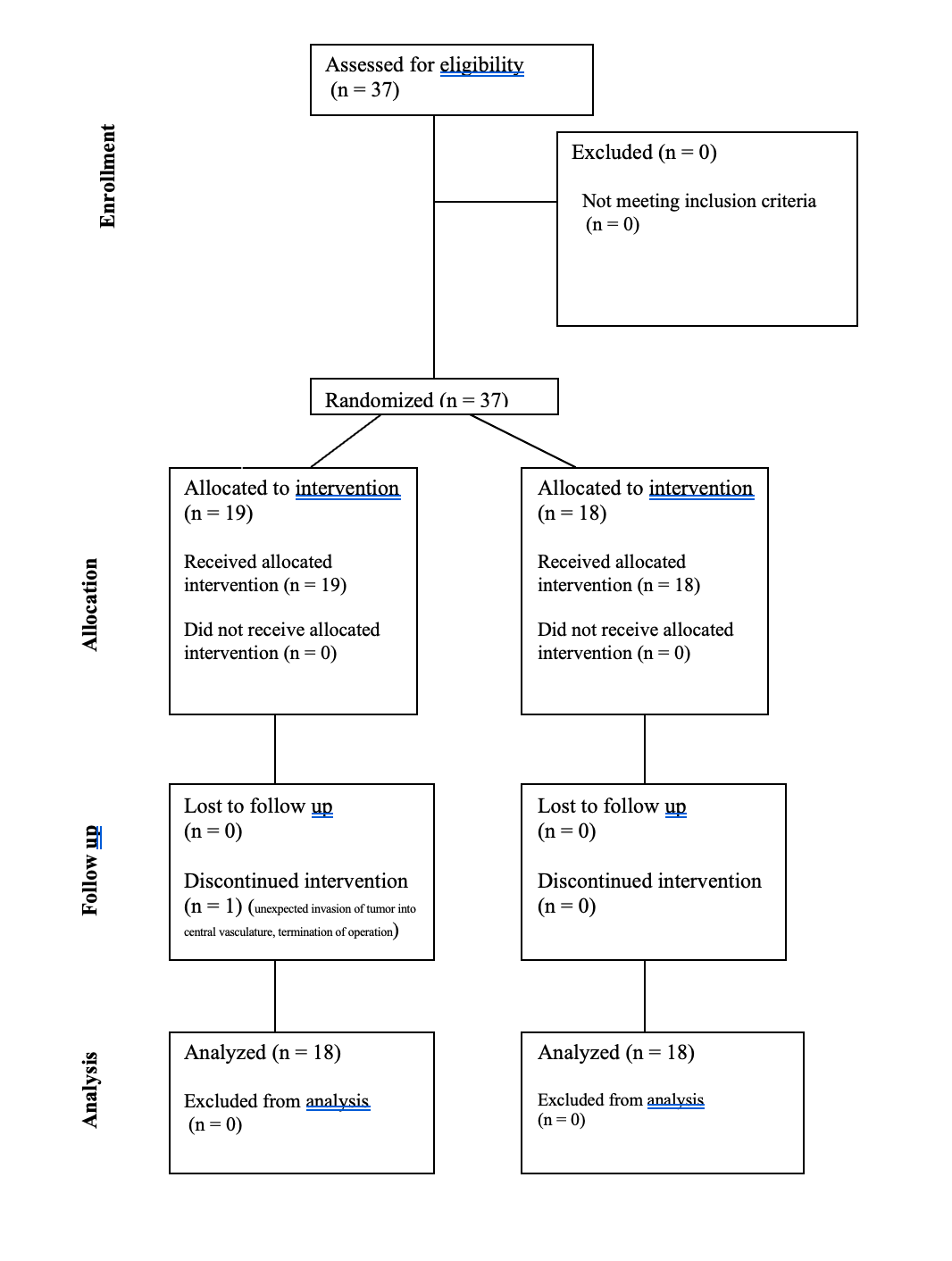
Additional references figure 5 – Patient flow chart


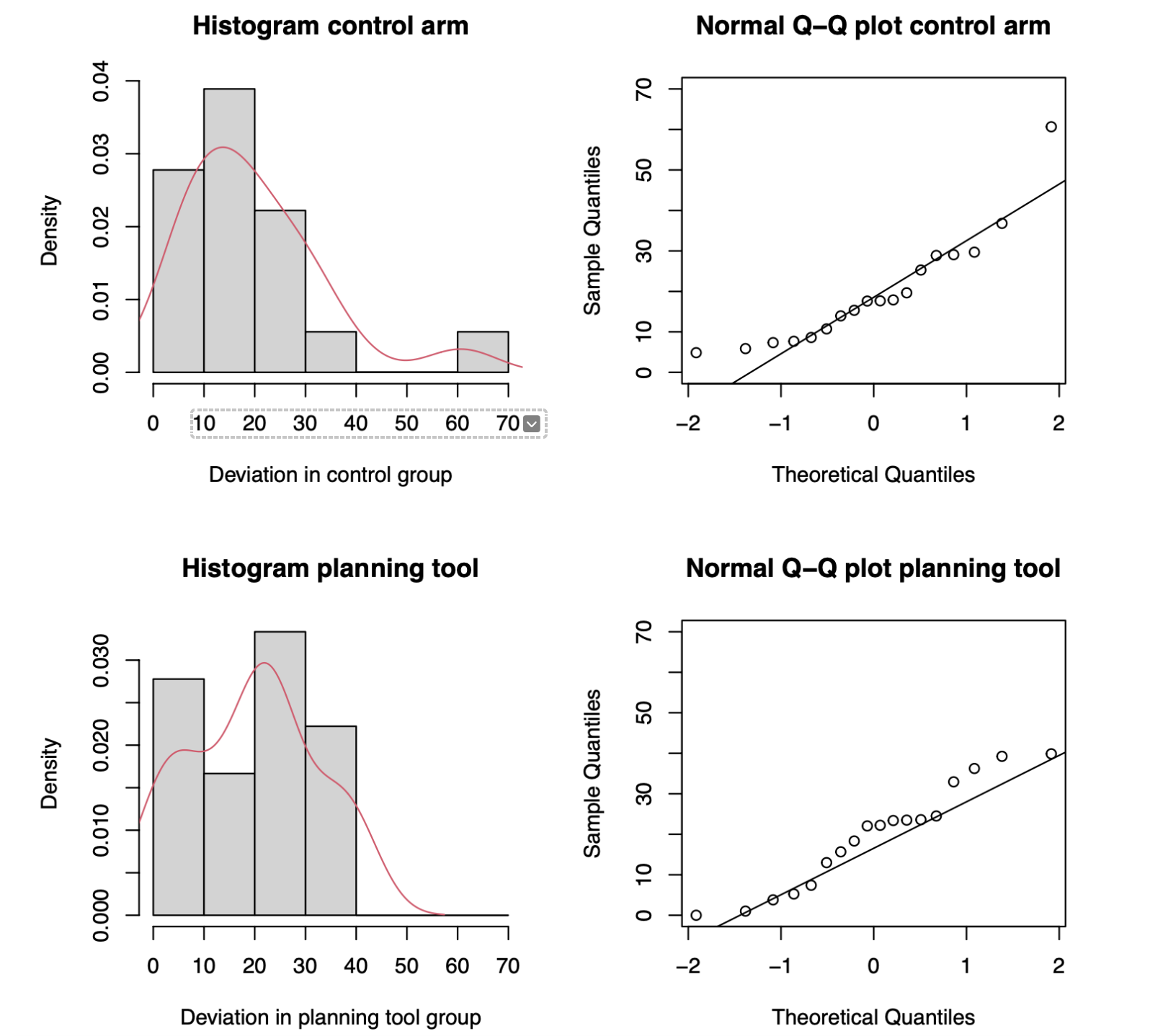


Additional references figure 6
